# Bending-Induced Progressive Damage of 3D-Printed Sandwich-Structured Composites by Non-Destructive Testing

**DOI:** 10.3390/polym17141936

**Published:** 2025-07-15

**Authors:** Lianhua Ma, Heng Sun, Xu Dong, Zhenyue Liu, Biao Wang

**Affiliations:** 1 Research Institute of Interdisciplinary Science & School of Materials Science and Engineering, Dongguan University of Technology, Dongguan 523808, China; 2 School of Quality and Technical Supervision, Hebei University, Baoding 071002, China; qschbu@163.com (H.S.); 15831256010@163.com (X.D.); 17835463796@163.com (Z.L.); 3 School of Physics and Sino-French Institute of Nuclear Engineering and Technology, Sun Yat-sen University, Guangzhou 510275, China

**Keywords:** fiber reinforced polymer, sandwich structures, acoustic emission, clustering analysis, micro-CT

## Abstract

With the extensive application of 3D-printed composites across multiple industries, the investigation into their structural reliability under complex loading conditions has become a critical research focus. This study comprehensively employs acoustic emission (AE) monitoring, digital image correlation (DIC) measurement, and micro-computed tomography (Micro-CT) visualization techniques to explore the progressive damage behavior of 3D-printed sandwich-structured composites reinforced with continuous carbon fiber sheets under three-point bending. Mechanical tests show that increasing the fiber content of face sheets from 10% to 20% enhances average bending strength by 56%, while low fiber content compromises stiffness and load-bearing capacity. AE analysis categorizes damage modes into matrix cracking (<50 kHz), debonding/delamination (50–150 kHz), and fiber breakage (>150 kHz) using k-means clustering algorithms. DIC measurement reveals significant structural deformation processes during damage progression. The AE-DIC-Micro-CT combination demonstrates an initial undamaged state, followed by damage initiation and propagation in the subsequent stages. This integrated approach provides an effective method for damage assessment, guiding the design and reliability improvement of 3D-printed composites.

## 1. Introduction

Fiber reinforced composites are excellent structural materials with exceptional mechanical properties, such as high specific stiffness, specific strength, and good thermal stability [1]. Therefore, fiber reinforced composites are widely used in aerospace, the auto industry, and other fields due to their superior mechanical properties [2]. Fiber reinforced composites can be fabricated by pultrusion molding, vacuum infusion, and filament winding technologies [3,4]. These traditional manufacturing methods usually suffer from a long manufacturing cycle time, high production costs, and complex processes [5,6].

The emerging three-dimensional (3D) printing technology has a significant advantage for the manufacturing of complex structural components. Different from traditional manufacturing methods, 3D printing technology is undergoing rapid development with a highly automated, low cost, and short production cycle [7]. The structural components can be fabricated quickly by using computer-aided modeling without the limitation of shape. Hence, this developing technique provides an opportunity to fabricate materials and structures with complex shapes [8]. However, a lower carrying capacity is a disadvantage of 3D-printed products. To expand the range of load-bearing applications, various scholars used fibers as reinforcements to improve the mechanical properties of 3D-printed products [9,10]. Melenka et al. [11] compared the tensile properties of specimens with different fiber contents and proposed a method to predict the tensile properties of 3D-printed samples. The study reveals a positive correlation between fiber content and both stiffness and strength. Another investigation conducted by Li et al. [12] evaluated the mechanical properties of carbon fibers and modified carbon fiber-reinforced composites. Their mechanical tests and SEM characterization show that the interfacial strength of modified carbon fiber specimens was improved. Chang et al. [13] evaluated the mechanical properties of 3D-printed CCF/PEEK samples subjected to bending and tensile loads. The effects of laser power and consolidation speed on the bending properties were studied and the optimal printing parameters were determined. The mechanical properties were further improved by hot pressing post-treatment. Masahito et al. [14] fabricated carbon fiber-reinforced thermoplastic (CFRTP) through 3D compaction printing and investigated the tensile and bending properties. The experimental results and microscopic analysis results revealed that 3D compaction printing improved the tensile and bending properties of the specimens and reduced porosity. Mei et al. [15] reported the mechanical properties of 3D-printed carbon fiber composites with mixed isotropic fiber angles and further analyzed the effects of different pressures, temperatures, and times on the hot press. Peng et al. [16] investigated the effects of the raster angles of continuous carbon fiber, stacking sequence, and loading direction on the mechanical behaviors of 3DP-SCCFRPA through a three-point bending test. The deformation process and damage mechanism of the specimens were analyzed with the help of SEM. The effects of fiber content, filling density, and fiber orientation on mechanical properties were evaluated by a tensile and bending test, uniaxial compression test, and impact test [17,18,19]. Similar to traditional manufacturing techniques, some process parameters need to be considered during the printing process [20,21,22]. To some extent, the printing parameters influenced the mechanical properties of the specimens. However, the characterizations of the internal damage process of a 3D-printed composite subjected to complex loads are seldom reported.

Due to the unique ability of 3D printing to fabricate complex geometries, it is necessary to adopt nondestructive testing technology to investigate the damage mechanisms of the printed specimens [23,24]. Barile et al. [25] studied the effects of different extrusion temperatures on inter-layer cohesion for ABS printing specimens, and dynamic monitoring was carried out with the help of acoustic emission technology. It was demonstrated that increasing the extrusion temperature reduces stratification while enhancing toughness. Zhang et al. [26] utilized micro-CT and DIC technology to investigate the damage mechanism of 3D-printed woven composite plates with a hole under tensile and shear loading. The result demonstrated the application of micro-CT and DIC provided a means to study the deformation and microstructural evolution of 3D-printed samples. He et al. [27] analyzed the effects of pores on the mechanical properties of CF/PA6 composites through one tensile and three bending experiments. In order to further reveal the internal damage mechanism, CT and SEM were used to characterize the microstructural morphology of the specimens. Somireddy et al. [28] theoretically and experimentally investigated the mechanical properties of short carbon fiber-reinforced specimens with different thicknesses. The failure mechanism of the specimens was further evaluated by micro-CT characterization. Shulga et al. [29] analyzed the mechanical properties of short glass fiber-reinforced composites by conducting mechanical experiments and micro-CT characterization. It was found that fiber orientation has a significant influence on tensile strength. Pan et al. [30] studied the progressive damage behavior and failure mechanism of 3D braided composites under bending loads by using AE and micro-CT, but they only examined the effects of different fiber types on flexural strength. Essassi et al. [31] prepared four kinds of sandwich-structured specimens with different densities via additive manufacturing technology and used AE technology to monitor damage initiation and propagation up to the failure of such materials. Wu et al. [32] employed 3D printing to fabricate T-beams and utilized AE monitoring to study the bending-induced failure process; they found that 3D-printed T-beams can significantly improve load-carrying capacity. Although the damage of 3D-printed composites was experimentally investigated, the damage evaluation was mainly determined by a single testing method. Some studies have combined micro-CT technology with acoustic emission or other techniques for joint analysis. However, these studies only conducted micro-CT inspections of the structural damage after failure, without achieving the visualization representation of the progressive damage process of the structure.

In summary, there was a lack of in-depth research on the damage evolution and failure mechanism of 3D-printed composite materials. To further characterize the damage characteristics, it is necessary to combine multiple non-destructive testing techniques to monitor the damage process of the structure. In this study, a combination of AE and DIC technology is used to monitor the failure process and deformation evolution in 3D-printed sandwich-structured composites subjected to bending loads. In addition, the internal damage evolution of the sample is characterized by progressive micro-CT imaging. To process the AE signals generated during bending tests, the k-means algorithm is used to perform a clustering analysis. Based on AE, DIC, and CT technology, the damage mechanisms and failure mode of 3D-printed sandwich-structured composites with different fiber contents under bending loads are systematically examined in this study.

## 2. Materials and Experimental Procedure

### 2.1. Materials and Specimens Fabrication

Based on the FDM technique, continuous fiber-reinforced specimens were manufactured by the Mark X7 printer, which is equipped with two nozzles offering the function of printing fiber-reinforced composites. The adopted printing material was an Onyx filament, which was supplied by the Markforged Corporation, Watertown, MA, USA. It is a mixture of nylon and cut fibers, and has a diameter of 1.75 mm. Continuous carbon fiber was used as the reinforcement phase and was also provided by the Markforged Corporation. Two kinds of sandwich-structured composite specimens with different fiber contents were fabricated using the Mark X7 3D printer. The testing specimens were both designed as sandwich structures with the dimension of 60 mm × 20 mm × 5 mm. The specimen geometry was first constructed through computer-aided software (Solidworks 2016), then exported as a stereolithography file (STL) for manufacturing with the 3D printer. For each type of 3D-printed sandwich-structured composite, a total of three specimens were used for testing. Two nozzles were used to print the matrix phase and the reinforcement phase of the FRP face sheets, respectively, during the manufacturing process. The matrix material from one nozzle was heated to 275 °C and the fiber phase from another nozzle was heated to 135 °C. Then, the sandwiched specimens were printed layer by layer based on the printing platform.

**Figure 1:**
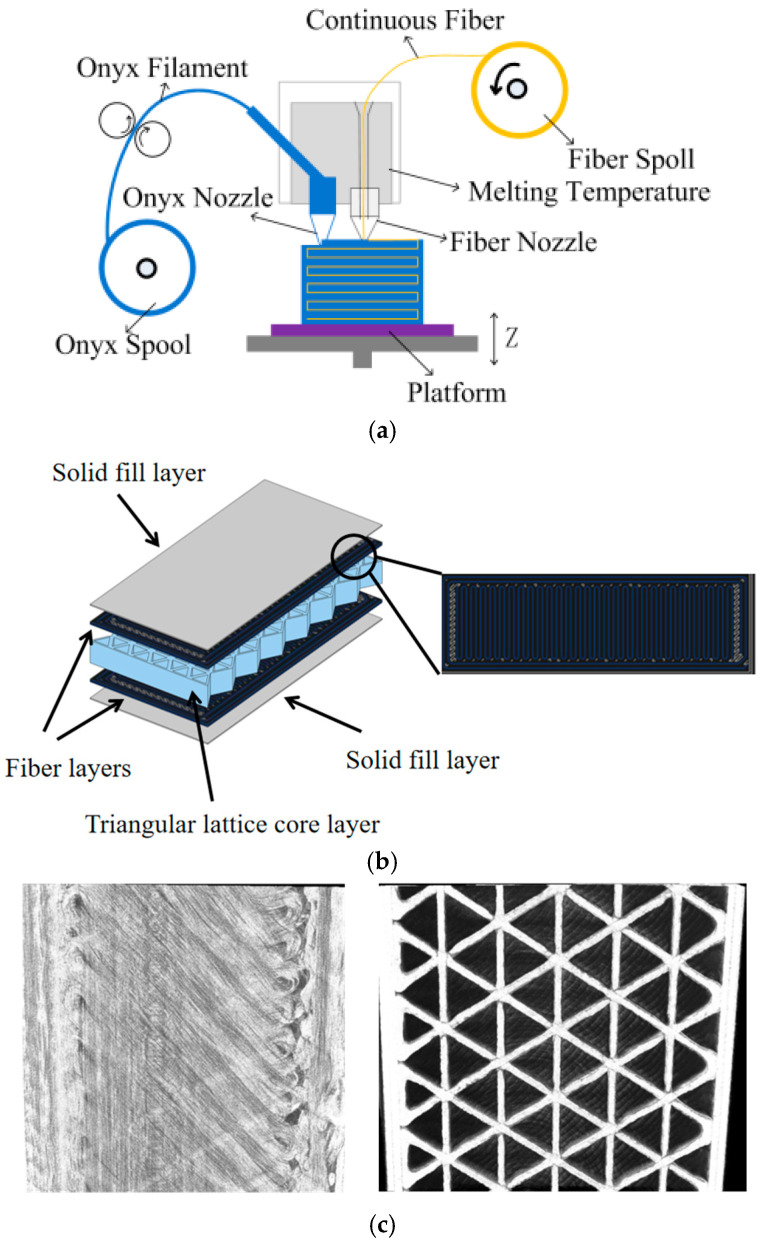
Schematic of (**a**) 3D printing process, (**b**) representative geometry of the sandwich structure, and (**c**) microstructure of the printed FRP face sheet and triangular lattice core of the sandwich-structured composite.

Figure 1 presents the schematic diagram of the 3D printing process, representative geometry of the sandwich structure, and the microstructure of the fiber-reinforced face sheet and triangular lattice core supporting layer of the sandwich-structured composite. The printed composite sandwich structure consists of two thin, strong face sheets, and two solid fill layers on the outer surface, with a lightweight, low-density triangular lattice core layer between the two face sheets, as sketched in Figure 1b. The face sheets are typically printed by continuous carbon fiber-reinforced polymer, while the core layer is constructed by a triangular lattice structure. The continuous carbon fibers endow the face sheets with superior strength and stiffness properties. By strategically combining a core supporting layer with carbon fiber sheets, the final sandwich-structured composite with a much higher strength-to-weight ratio can be printed. By varying the fiber content of the face sheets, different FRP sandwich composites can be constructed. For the purpose of comparison, two types of composite specimens with different fiber contents were printed. The adopted printing parameters of the two types of specimens are listed in Table 1. Each specimen consists of 4 onyx layers on the top, 4 onyx layers on the bottom, and 2 vertical layers. The microstructures of the printed FRP face sheets and the lattice core layer of the sandwich composite were characterized by micro-CT scanning, as depicted in Figure 1c.

### 2.2. Mechanical Testing and Characterization

The mechanical properties of the CFRP sandwich specimens were studied by three-point bending tests, which were carried out on an experimental machine (LD24) according to the ASTM D790 standard [33]. The specimens were tested at a strain rate of 1 mm/min, while maintaining a length of 48 mm between the supporting rollers. To real-time monitor the damage process of the specimens, AE technology was used to identify the damage modes during the loading process. AE signals were collected by two broadband sensors (RS-54A), which were affixed on the surfaces of the specimens. The set frequency ranged from 100 to 900 kHz and the distance between the two AE sensors was 40 mm. To improve the acquisition effect of the AE signals, silicone grease was coated on the surface of the AE sensors. To reduce the influence of noise from the machine and the surrounding environment, the acquisition threshold was set as 10 mv (40 dB) through tentative operations. The sampling frequency was set to 3 MHz, while the peak definition time (PDT), hit definition time (HDT), and hit lockout time (HLT) were configured to be 30 μs, 150 μs, and 300 μs, respectively.

The DIC system equipped with a metal-oxide-semiconductor (CMOS) camera (DaHeng Imaging, Shenzhen, China) was used to measure the displacement and strain of the specimens during the loading process. The adopted camera has 2592 × 1944 pixels resolution, capturing two pictures per second [34]. The fractured specimens were scanned by a micro-CT system (Skyscan 1172, with a resolution of 6.8 μm, an acceleration voltage of 75 kV, and a current of 132 μA) to identify the morphology features. The platform was operated by rotating at a fixed increment to acquire slices from different angles. Subsequently, the obtained CT images are processed and reconstructed by image processing software (Nrecon V1.6.9.8 and Ctvox 2.1). The damage morphology was analyzed and studied through reconstructed images.

## 3. Results and Discussion

### 3.1. Mechanical Properties

The mechanical properties of the specimens are investigated through a series of three bending experiments. The average failure loads of specimens A and B are 595.03 and 383.16 kN, respectively, with corresponding standard deviations of 15.46 and 33.99 kN. According to Equation (1), the computed flexural strengths of specimens A and B are 85.68 MPa and 55.17 MPa, respectively.

(1) $$S=\frac{3\times P\times L}{2\times w\times h^{2}}$$

where w (mm) denotes the width of the specimens, *h* (mm) is the thickness, *P* is the maximum load, and *L* is the support span.

The results of the failure load and flexural strength of the two kinds of composites indicate that an increasing fiber volume fraction results in an increase of the failure load and flexural strength. This is attributed to the fact that continuous fibers are the main load-bearing phase for the composite specimens.

**Figure 2:**
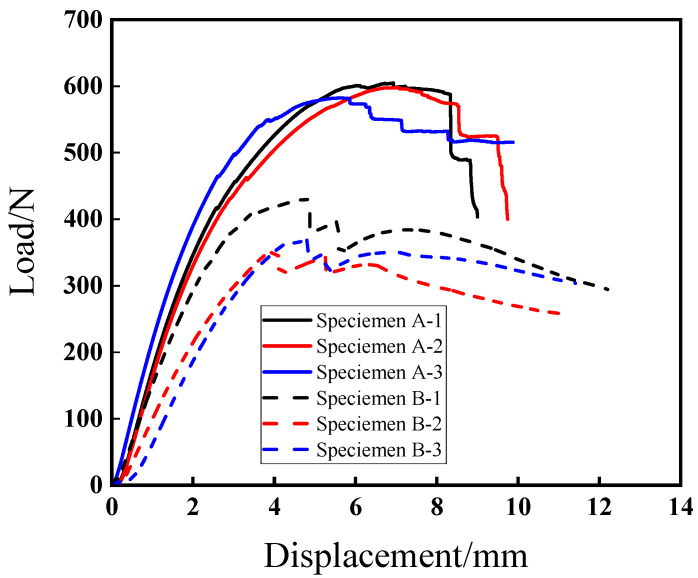
Flexural load–displacement curves of two types of 3D-printed composite specimens.

It is well accepted that fiber content plays an important role in the mechanical properties of 3D-printed composites. Figure 2 plots the typical displacement–load curves of specimens A and B with different fiber volume fractions. For each type of specimen, the displacement–load curves have similar variation trends. With an increase of the fiber content, the flexural strength and rigidity of the composites are increasingly enhanced. For both specimens A and B, there is an obvious linear elastic behavior during the initial loading stage. When the load increases to some extent, the curves shift to the inelastic stage. It is readily apparent that the loading curves corresponding to specimen A exhibit a dramatic downward trend after reaching the maximum load in comparison with specimen B, which indicates that the toughness of the specimens with a higher fiber content decreases due to fiber breakage [34]. To further reveal the deformation and progressive damage mechanism of such 3D-printed composites, AE technology was used to monitor the damage and failure process during the three-point bending test.

### 3.2. AE Monitoring and Signal Analysis

**Figure 3:**
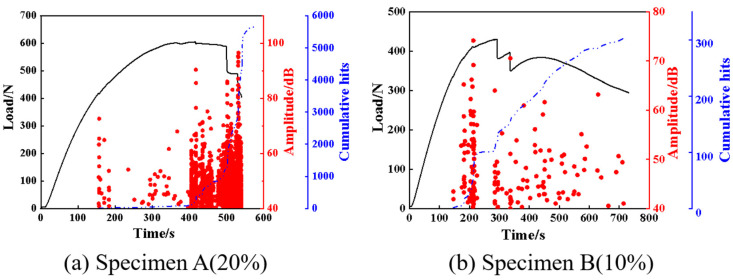
Load, amplitude, and cumulative hit evolutions with time for specimens A and B.

**Figure 4:**
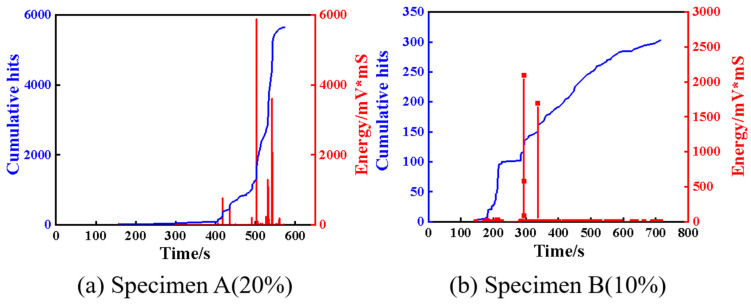
Energy and cumulative evolutions with time for specimens A and B.

AE is a technology capable of real-time monitoring of internal damage in composite materials. The damage evolution and damage mode of the investigated specimens in this study can be analyzed by acquiring AE signals in bending experiments. It is well known that the generation of AE signals is accompanied by the accumulation of damage and the release of energy [35]. Therefore, the analysis of AE parameters such as amplitude, cumulative hits, energy release, and peak frequency is important to identify the damage modes of composite materials. For the AE data generated in the experiments, two groups of representative data were selected for signal analysis. In the AE experiment settings, the effect of ambient noise was eliminated by adjusting the threshold value. Figure 3 illustrates the typical loading curves, amplitude, and cumulative hits with time for both specimens. It can be observed that no AE signals are generated in the initially elastic stage. When the specimens deform into the plastic stage, AE signals are produced and the cumulative hits gradually become larger, which manifests micro-cracks that are initially formed inside the specimens. The AE signals and the cumulative hits increase significantly near the maximum load, which can be attributed to damage accumulation and the propagation of the specimens. When the loads approach the final failure value, the AE signals and the cumulative hits nearly reach their maximum, arising from the occurrence of multiple damage modes during the failure process, such as matrix cracking, delamination, and fiber breakage. Compared to specimen A, the AE signals of specimen B are somewhat different. The cumulative hits of specimen B increased rapidly at about 200 s, while those of specimen A started to increase rapidly at about 500 s. This may be attributed to the early failure of specimen B. Figure 4 depicts the relationship among the cumulative hits, energy, and time. It can be observed from Figure 3a and Figure 4a that the composite material exhibits no discernible damage and the AE energy is relatively low during the initial loading stage of the specimen. With the increase of the load, some high energy AE signals were produced, indicating the specimen had been damaged. At the point of failure, both the cumulative hits and the released energy increase sharply. The energy value reaches 6000 mV*mS, which further implies that the specimen was seriously damaged. For specimen B, a high energy signal is produced at about 300 s. This suggests that specimen B suffered from serious damage near this position while bearing most of the load. In addition, as observed in Figure 4, the energy peak of specimen B was much smaller than that of specimen A. The damage evolution of the composites may be preliminarily illustrated by analyzing the AE characteristic parameters, but they are insufficient to identify the intrinsic damage mechanism. Therefore, a clustering analysis of AE is proposed to further reveal the damage mode of the composites.

Due to the complexity of the relationships between individual AE parameters, damage mode identification relies mainly on supervised or unsupervised clustering methods. k-means clustering serves as an unsupervised iterative algorithm based on minimizing the distance between each data point and its corresponding cluster centroid. This method is efficient and has strong interpretability of clustering results, making it widely adopted for distinguishing damage modes in composites. Typical applications include failure sequence determination in sandwich structures using concurrent acoustic emission monitoring and postmortem thermography [36], as well as analyzing the cryogenic damage behavior of carbon fiber-reinforced polymer composite laminates via fiber-optic acoustic emission [30,37]. The Davis–Bouldin index and Silhouette index are used as evaluation indexes for clustering quality, and the optimal cluster number of k-means clustering is determined to be three. Therefore, to roundly identify the damage mechanism of 3D-printed composites, two important characteristic parameters, the peak frequency and amplitude, are analyzed by the *k*-means algorithm to cluster AE signals.

**Figure 5:**
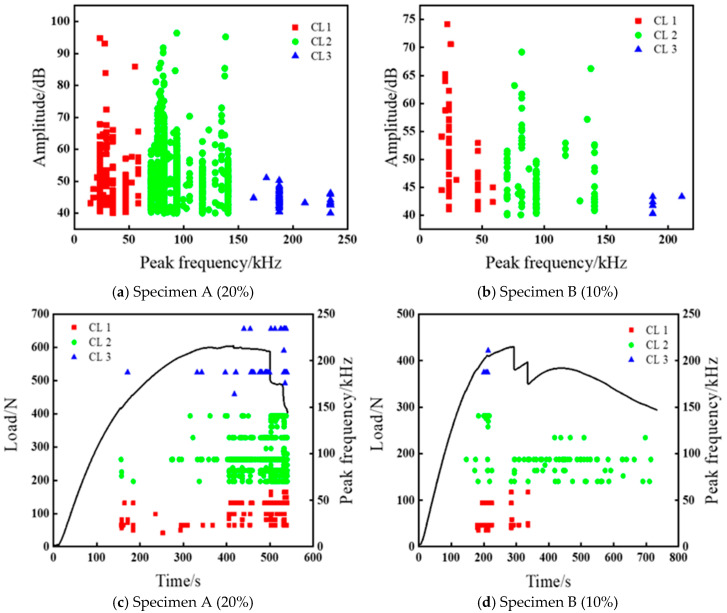
(**a**,**b**) Relationship between amplitude and peak frequency for specimens A and B. (**c**,**d**) The load and frequency evolutions with time for specimens A and B.

Different peak frequencies are associated with different damage modes [38]. Figure 5 shows the amplitude and frequency distribution of three typical damage modes of the 3D-printed composite specimens. As can be seen from the figure, the boundaries between each cluster are relatively clear, indicating a high level of clustering quality. Based on the statistical analysis, the AE signals can be clustered into three categories: CL 1: 17–65 kHz (40–94.8 dB), relating to the matrix cracking; CL 2: 70–150 kHz (40–96.3 dB), corresponding to crack growth or fiber delamination; and CL 3: >150 kHz (40–57 dB), representing fiber breakage [39]. It can be seen from Figure 5a,b, that the amplitude distribution of fiber breakage for the two types of specimens is smaller than that of matrix cracking and fiber/matrix debonding.

The abundance of AE signals indicates that the fiber/matrix debonding is the predominant damage mode in 3D-printed fiber-reinforced composites. All the AE signals relating to fiber fracture have the highest peak frequencies, but the amplitude is lower than that of matrix cracking and fiber/matrix debonding, and this result is consistent with the findings of Zhou and Zhang [35,40]. The damage modes of 3D-printed continuous carbon fiber-reinforced composites under bending loads are mainly assigned to matrix cracking, fiber/matrix debonding, and fiber fracture. The occurrence of matrix cracking is associated with low-frequency and high-amplitude AE signals, while fiber/matrix debonding and delamination are characterized by medium-frequency AE signals. Fiber fracture is indicated by high-frequency and low-amplitude AE signals [41]. In addition, Liu et al. [42] also verified the above frequency features by designing experiments with a single damage mode and adopting a cross-scale recognition method based on deep learning. The loading curve and the acquired frequency evolution of the 3D-printed composites during the bending deformation are shown in Figure 5c,d. It can be readily observed that the initial damage stage of specimen A corresponds to the low-frequency signals, indicating that the damage is primarily induced by matrix cracking. With the load increases, medium- to high-frequency signals are gradually generated, implying that multiple damage modes occur at this stage. Due to the lower fiber content of specimen B, the AE signal mainly corresponds to medium and low frequencies. Matrix cracking and fiber fracture contribute to the main sources of the AE signals of such composites. Therefore, with the increase of the fiber volume fraction, the AE signals are gradually getting richer. The damage mechanism of the specimens can be fully characterized by a cluster analysis of the main characteristic parameters of AE signals.

### 3.3. DIC Measurement

The DIC method is employed to measure the strain fields of 3D-printed composites subjected to bending loads. Displacement and strain under a bending load can be calculated using the algorithm of the maximum correlation coefficient C [43]. With the increase of the selected interval, the correlation of images decreases. Consequently, a photograph was chosen at an interval of 50 N to calculate the strain fields of the specimens.

**Figure 6:**
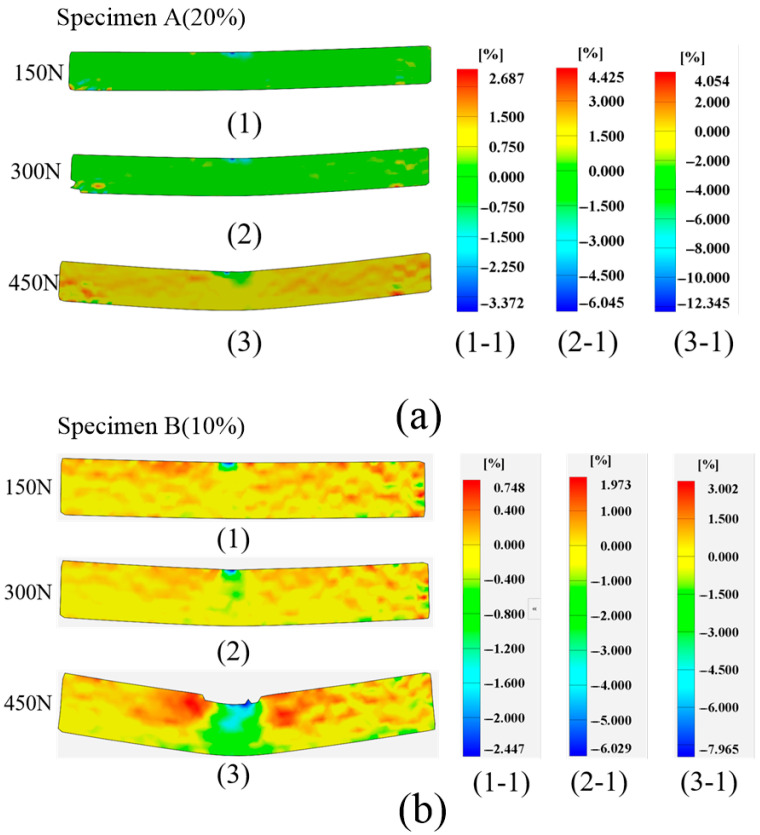
Strain field distribution for specimen A and B. (**a**) Vertical strain for specimen A, (**b**) Vertical strain for specimen B.

Figure 6 shows the vertical strain field distribution for both specimens A and B under different load levels. After subjection to different loading levels, the maximum strain magnitudes of specimen A are 3.372%, 6.045%, and 12.345%. For comparison, the maximum strain magnitudes of specimen B are 2.447%, 6.029%, and 7.965%, respectively. The apparent strain concentration can be found in specimen B when the load increases to 450 N, as depicted in Figure 6b. On this occasion, the released energy was monitored to sharply increase, as shown in Figure 4b, implying that remarkable deformation and damage are generated. Hence, the combination of the AE and DIC technologies enables a complementary understanding of the deformation and damage in 3D-printed composites. Although these two techniques can be effectively used to monitor the deformation and damage of composites, they cannot directly characterize the internally damaged morphology. Therefore, further investigation into the modes of damage of composites is imperative.

**Figure 7:**
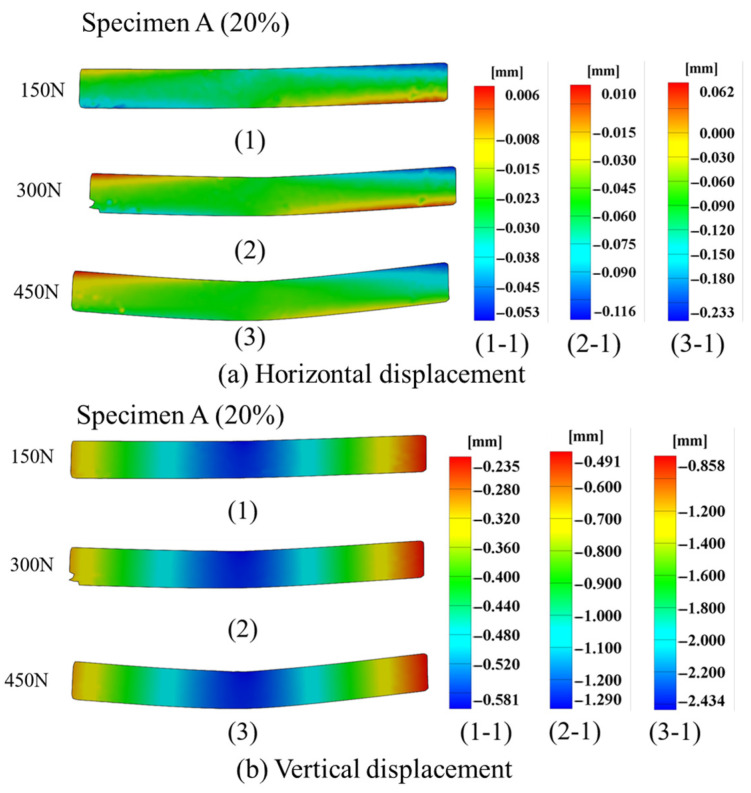

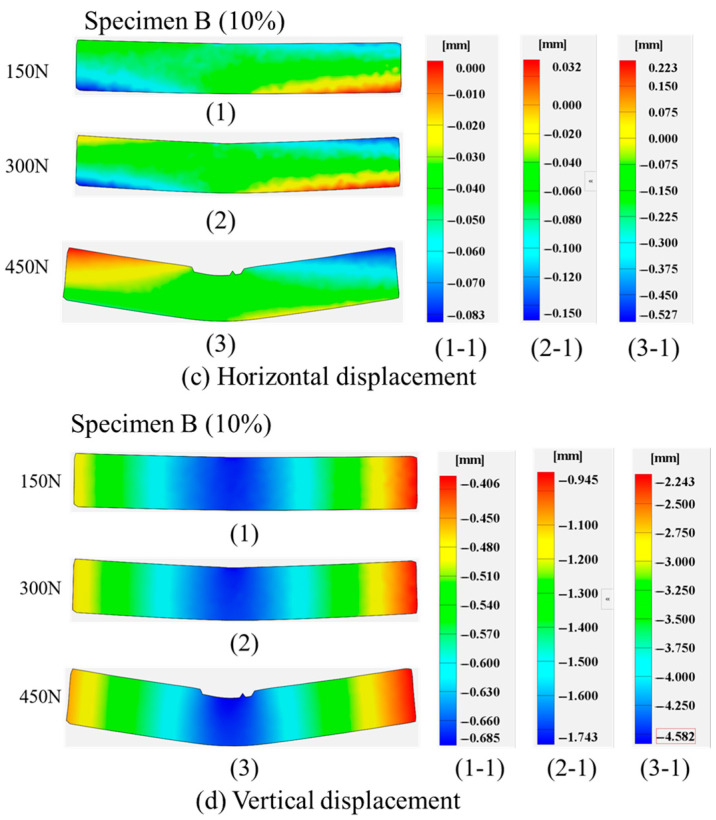
Displacement field distribution for specimens A and B.

When the load increases from 150 N to 450 N, Figure 7 shows the displacement field distribution of the 3D-printed composites with different fiber volume fractions. The vertical displacement of the composite specimens was larger than the horizontal displacement, with maximum vertical displacements of 2.434 mm and 4.582 mm, respectively, as illustrated in Figure 7. As observed in Figure 7b,d, the vertical displacement increment of specimen A was smaller than that of specimen B under identical loading conditions. This phenomenon indicates that specimen A exhibits higher stiffness than specimen B. As the load increased to 450 N, the displacement field of specimen B showed missing data points. This occurrence signifies that specimen B failed at 450 N due to excessive deformation, rendering an accurate displacement field calculation impossible. In general, large deformation serves as an indicator of specimen failure. Simultaneously, this phenomenon correlates with the increase in AE signals and the cumulative hits of specimen B shown in Figure 3.

### 3.4. Progressive Damage Analysis

**Figure 8:**
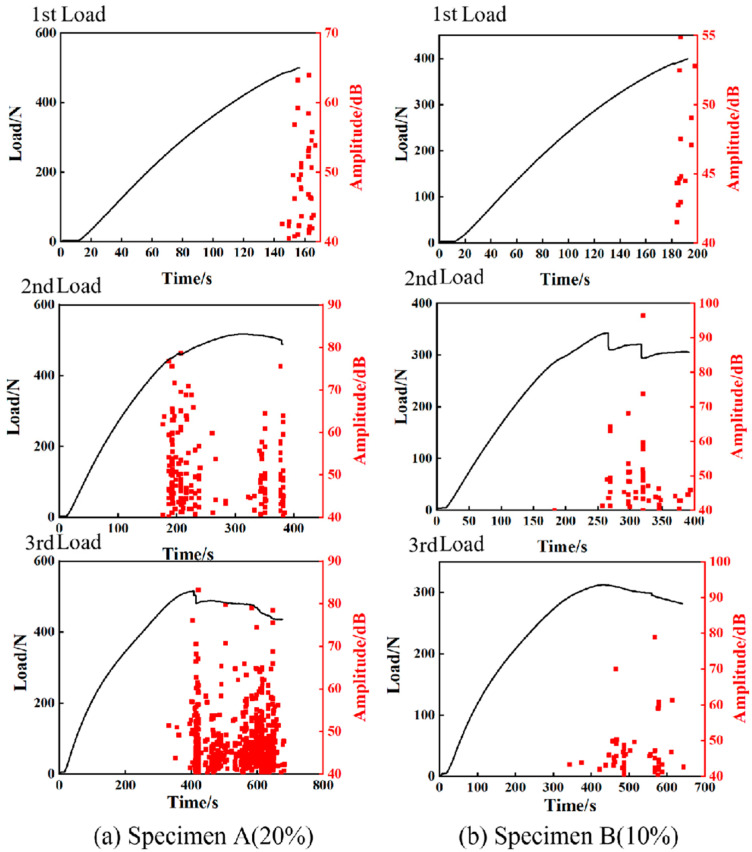
Flexural load and amplitude of specimens A and B under three loading stages.

**Figure 9:**
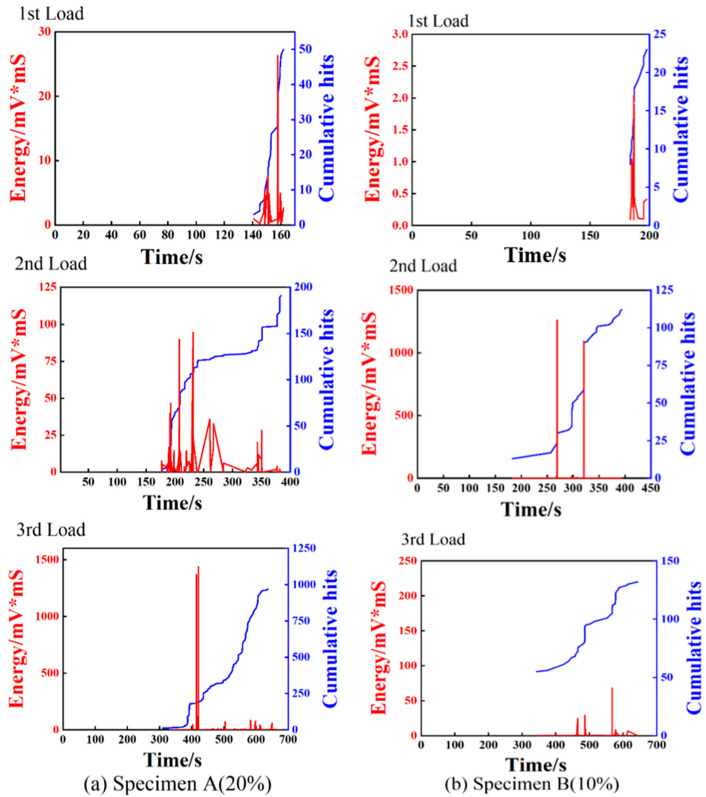
Energy and cumulative hits of specimens A and B under three loading stages.

To further reveal the damage mechanism of 3D-printed composites under different bending loads, a progressive damage analysis was carried out based on AE and micro-CT characterization. Three typical bending loading levels were respectively applied on the composites for a damage analysis by stages. For the two types of specimens under three loading stages, the evolutions of load, amplitude, energy, and cumulative hints extracted from the AE signals with loading time are given in Figure 8 and Figure 9.

For the first loading stage, the AE signals are produced at the loading occasion of about 500 N for specimen A, while the signals are detected at about 400 N for specimen B, indicating that specimen A has a relatively large carrying capacity due to its large fiber content. As illustrated in Figure 9, the energy and cumulative hits of specimen A are higher than that of specimen B, indicating that specimen A undergoes worse damage after the first loading stage. This can be attributed to the large fiber content of specimen A leading to more obvious brittle failure. For the second loading stage, the specimens were loaded to undergo a nonlinear deformation state. As observed from the AE characteristics shown in Figure 8 and Figure 9, no distinct AE signals are detected until the first loading level is reached, which is consistent with the so-called Kaiser effect. With the increase of the loading, the AE signals are getting richer, due to the generation of multiple damage modes such as matrix cracking, fiber debonding, and delamination. In addition, the recorded AE energy values of the two specimens are significantly different, arising from different damage modes in the two composites. Finally, the third group of loading experiments were carried out to bend the specimens to reach final failure. As shown in Figure 8, obvious nonlinear deformation characteristics can be observed for both specimens, indicating that multiple damages are progressively produced before the catastrophic failures. The AE energy peak and cumulative hits of specimen A are considerably higher than those of specimen B, as illustrated in Figure 9. This demonstrates specimen A undergoes more intense damages than specimen B during the loading process, which is consistent with the evolutions of the event amplitude content of the AE signals for the specimens shown in Figure 8. This is because the composite with a higher fiber content exhibits weaker toughness and tends to evoke brittle failure.

**Figure 10:**
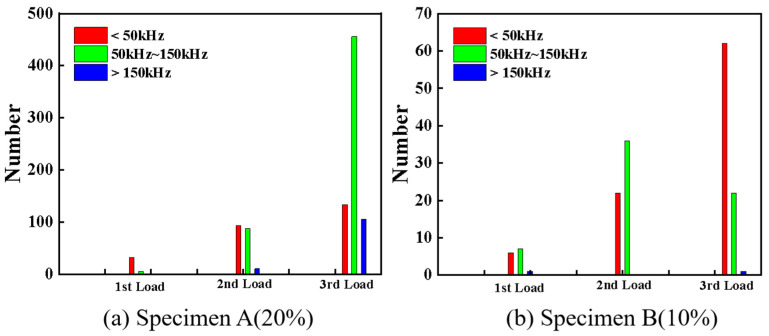
The frequency distribution of specimens A and B in three loading stages.

As an integral evaluation, by combining the AE signals for the three loading stages, the frequency distributions of the two types of specimens are presented in Figure 10. It is readily observed that the AE events of specimen A are more abundant than those of specimen B for almost all the damage modes produced during the three loading stages. For the first loading level, the damage modes of specimen A are dominated by matrix cracking corresponding to 0–50 kHz frequencies, while the matrix cracking and delamination corresponding to 50–150 kHz frequencies are both dominant in specimen B. In addition, a few AE signals with high frequencies (>150 kHz) can be observed during the first and second loading stages for specimen A and all the loading stages for specimen B, indicating very slight fiber breakage can be detected in these cases. However, fiber breakage is prominent during the final load stage of specimen A. During the second loading stage, both the matrix cracking and delamination in the FRP sheets are the dominating damage modes in the two types of sandwich structures, but the delamination damage is more remarkable in the specimen with a high fiber content. For the third loading stage, different damage modes can be identified in the two specimens. Delamination and matrix cracking are the predominant damage modes of specimens A and B, although the three types of damage signals are detected in both the specimens. It is worth noting that the AE events corresponding to the fiber breakage monitored in specimen B are much less than those of specimen A. It can be seen from the frequency distribution presented in Figure 10 that the fiber content of the sheets has a significant effect on the damage models of the 3D-printed sandwich structures under different load levels.

**Figure 11:**
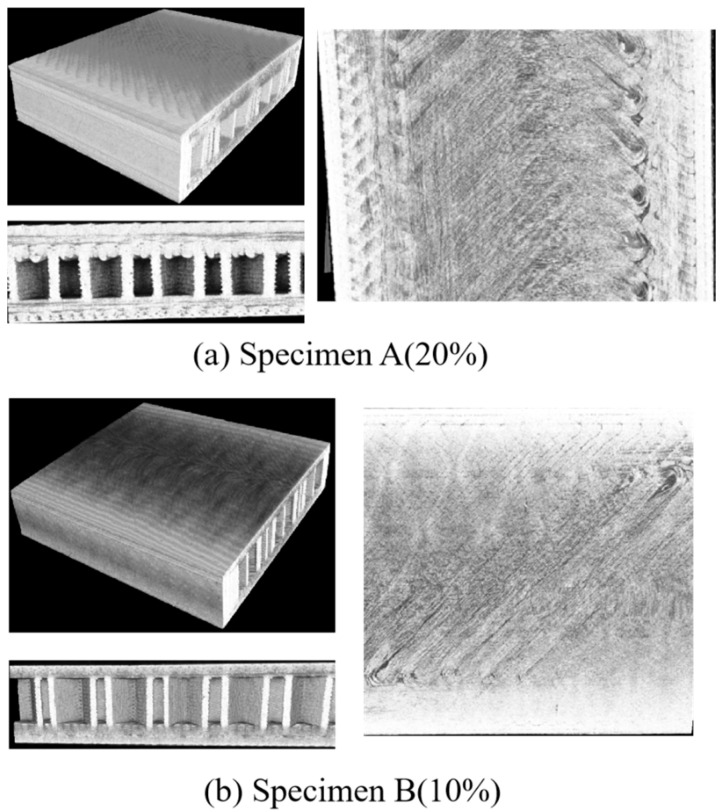
The reconstruction images and damage morphology of the sandwich specimens after the first loading stage.

**Figure 12:**
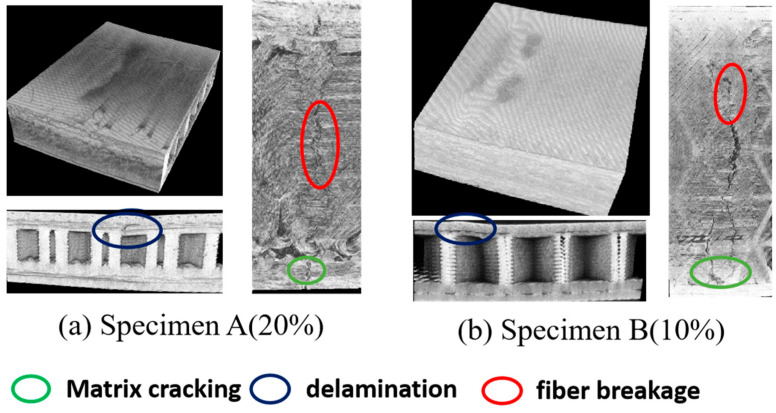
The reconstruction images and damage morphology of the sandwich specimens after the second loading stage.

**Figure 13:**
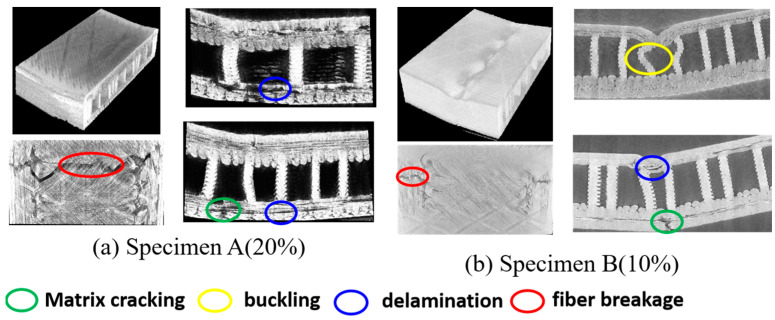
The reconstruction images and damage morphology of the sandwich specimens after the third loading stage.

Micro-CT is an effective method to visualize the damage morphology of 3D-printed sandwich samples at the micro level. To intuitively determine the internal damage patterns of the sandwich structure, the 3D-reconstructed micromorphology and cross-section views are given in Figure 11, Figure 12 and Figure 13, corresponding to the three load levels. As observed in Figure 12, no obvious damage patterns are observed in the two types of sandwich specimens, which is consistent with the monitored low AE signals mentioned above. This indicates that maybe only microcracks are initiated during the early load stage. However, the typical damage patterns including matrix cracking, delamination, and fiber breakage of the FRP sheet can be clearly observed after the second load stage, as shown in Figure 12. Evident indentations arising from unrecoverable plastic deformation and damage can be observed on the surfaces of the two types of specimens. It is noted that, compared to specimen A, specimen B undergoes more serious damage due to the lower fiber content of its sheets. After the third loading stage, both the specimens are directly loaded to catastrophic failure, and the more serious damages are presented in Figure 13. It is readily observed that the delamination damages are more remarkable than the matrix cracking in specimen A, while the matrix cracking damages are much more serious in specimen B, which agrees well with the frequency analysis of the AE signals from Figure 10. Fiber breakages are observed in both the specimens, but they are more notable in specimen A due to its higher fiber content. In addition, the sandwiched core structures exhibit dramatic buckling behaviors in the vicinity of the loading position on specimen B, while no distinct core buckling is observed in specimen A. We attribute this to the fact that the abundant fibers in the FRP sheets of specimen A bear more tensile stress than specimen B, and the compressive loads acting on the middle supporting core layer in specimen A are not large enough to induce micro buckling.

## 4. Conclusions

In this study, the progressive damage behavior and evolution mechanism of 3D-printed sandwich-structured composites with continuous carbon fiber-reinforced sheets subjected to three-point bending loads were experimentally investigated by a combination of AE monitoring, DIC measurement, and micro-CT visualization. AE is used to real-time track and monitor progressive damage evolution, DIC allows for full-field strain measurements, and micro-CT is enabled to characterize the internal damage morphology of the 3D-printed sandwich structures.

The mechanical test results show that the average bending strength of the 3D-printed sandwich structure increases by 56% when the fiber content of the face sheet increases from 10% to 20%, and a low fiber content leads to reductions in stiffness and load-carrying capacity. The cluster analysis results from the AE signals show that the damage modes of the sandwich composites can be divided into three types: matrix cracking (<50 kHz), debonding and delamination (50–150 kHz), or fiber breakage (>150 kHz). The DIC-based full-field strain measurement results show that the composite structures sandwiched by FRP sheets with different fiber volume fractions deform significantly when multiple damages generate. According to the AE signals and micro-CT visualization of the sandwich-structured specimens, no obvious damages are produced in the early loading stage. The multiple damages (matrix cracking, interfacial debonding, and fiber breakage) are gradually initiated and propagated from the second to the third loading stage, and the underlying progressive damage mechanisms are revealed by the AE signals and micro-CT characterization. Furthermore, the effects of the fiber content of the FRP facet on the damage evolutions of the structure are examined. The cross validation of the AE analysis and micro-CT visualization illustrates the progressive damage evolutions of the 3D-printed sandwich panels with different fiber volume fractions under three-point bending well. The combination of the non-destructive testing techniques of AE, DIC, and micro-CT provides an effective method for the damage assessment and integrity monitoring of 3D-printed composites.

## Figures and Tables

**Table 1:** The 3D printing parameters of the carbon fiber sandwich-structured composites.

Parameters	Specimen A	Specimen B
Fiber content	20%	10%
Thickness	0.1 mm	0.1 mm
Lattice core	Triangle	Triangle
Packing density	37%	37%
Fiber layers	8	4

## Data Availability

The data will be available from the corresponding authors upon reasonable request.
